# Prevalence of astrovirus, adenovirus, and sapovirus infections among Iranian children with acute gastroenteritis 

**Published:** 2020

**Authors:** Seyed Dawood Mousavi Nasab, Fatemeh Zali, Hooman Kaghazian, Mohammad Reza Aghasadeghi, Rajab Mardani, Latif Gachkar, Abbas Ahmadi Vasmehjani, Nayebali Ahmadi, Ali Ghasemzadeh

**Affiliations:** 1 *Department of Research and Development, Production and Research Complex, Pasteur Institute of Iran *; 2 *Viral vaccine research center, Pasteur Institute of Iran, Iran*; 3 *Department of Clinical Biochemistry, Faculty of Medicine, Tehran University of Medical Science, Tehran, Iran*; 4 *Department of Hepatitis, AIDS and Blood-borne diseases, Pasteur Institute of Iran, Tehran, Iran*; 5 *Department of Viral vaccines, Pasteur Institute of Iran, Tehran*; 6 *Infectious Diseases and Tropical Medicine Research Center, Infectious Diseases Department, School of Medicine, Shahid Beheshti University of Medical Sciences, Tehran, Iran *; 7 *Department of Virology, School of Public Health, Tehran University of Medical Sciences, Tehran, Iran*; 8 *Proteomics Research Center, Faculty of Paramedical Sciences, Shahid Beheshti University of Medical Sciences, Tehran, Iran*; 9 *Gastroenterology and Liver Diseases Research Center, Research Institute for Gastroenterology and Liver Diseases, Shahid Beheshti University of Medical Sciences, Tehran, Iran*

**Keywords:** Gastroenteritis virus, Astrovirus, Adenovirus, Sapovirus, Polymerase chain reaction

## Abstract

**Aim:**

This study aimed to determine the prevalence of Human Astroviruses (HAstVs), enteric Adenoviruses (HAdVs), and Sapoviruses (SaVs) in acute diarrhea patients, as well as their relation to age, sex, and season.

**Background:**

Acute gastroenteritis is one of the most common diseases affecting children <5 years old and viral agents with approximately >75% are the major causative agent of acute infectious diarrhea. After Rotavirus and Norovirus, the greater viral agents of acute gastroenteritis include HAstVs, HAdVs, and SaVs. To the best of our knowledge, there are sparse studies in Iran detecting at least three enteric viruses as causative agents of diarrhea simultaneously.

**Methods:**

The sample was collected from children referring to pediatric medical centers in Tehran, Iran; they were tested for Astrovirus, enteric Adenovirus, and Sapovirus by conventional PCR method. The association of incidence of viral enteric agents was evaluated with age, sex and seasonal pattern in children <5 years old.

**Results:**

The positive case number among acute gastroenteritis patients was 17/120 (14.1%). Patients ranged in age within 1–60 months, but 52.9% were aged ≤ 12 months. Males comprised the majority (70.6), and the male: female ratio was 2.4. HAstV was the most frequently detected virus (6.7%), while SaVs were detected only in 2.5% of cases. Mixed infections were not detected in these samples. The highest rate of HAstV was identified in winter (66.7%), HAdV in fall (66.7%), and SaV in winter (33.3%).

**Conclusion:**

These findings underscore the importance of monitoring the epidemiology of HAstV, HAdV, and SaV as causative agents of viral diarrhea infections.

## Introduction

 Acute gastroenteritis (AGE) is one of the most common diseases affecting children <5 years old and leading to increased morbidity and mortality worldwide especially in developing countries (1). It is estimated that acute gastroenteritis causes more than 700,000 deaths annually to those 2 years of age where virus agents with approximately >75% is the major culprit of acute infectious diarrhea (2, 3). Among the major causative viral diarrhea, the Rotavirus, Norovirus and then human Astrovirus, enteric Adenovirus, and Sapovirus have been the major viral agents of acute gastroenteritis. Human adenoviruses (HAdVs) belong to family *Adenoviridae,* genus *Mastadeno virus *often causing a wide range of disease symptoms as the second or third agent of infantile diarrhea (4, 5). The enteric serotypes of HAdVs have been reported to be the cause of 2–31% in developing countries (6). Human Astroviruses (HAstVs), of the family *Astroviridae*, have been reported to be a leading cause of 2.3% to 8.9% in the developed country, while the average incidence worldwide is 11% (7-9). HAstV has been increasingly identified as an important agent of acute gastroenteritis in children, predominantly in children younger than 2 years of age and elderly (8, 10). Sapoviruses (SaVs ( belongs to the *Caliciviridae *family classified into five genogroups (GI-GV). According to studies, SaVs were detected in 2.7% to 15.4% of hospitalized children and from 3.7% to 19.2% of in outpatients with acute gastroenteritis. Although the reported outbreak numbers are less for SaVs than for other viral diarrhea,  SaVs have recently been reported as and usually under the age of 5 years (11-13). In developing countries including Iran, little information has been reported to determine the prevalence of HAdVs, SaVs, and HAstV. This study aimed to evaluate the prevalence of HAdVs, SaVs, and HAstV in acute diarrhea, and also to examine the association of incidence of viral enteric agents with age, sex and the seasonal distribution pattern in those presenting to pediatric medical centers in Tehran, a metropolitan city in Iran. 

## Methods

**Sample collection and viral RNA/DNA extraction**

Fecal samples were obtained from archived stool collected during 2014 to 2015 from children less than 5 years old referring to pediatric medical center in Tehran, Iran. The study protocol was approved by the Ethics Committee of Tarbiat Modares University (ethical code 52/5140). Fecal specimens were documented to be free of leukocytes, red blood cells and as well as and common bacterial and parasite pathogens. Diarrhea was defined according to the WHO criteria for children as the occurrence of three or more loose, liquid, or watery stools within 24 hours. For each case with acute infectious diarrhea, a form was completed to record the necessary demographic data such as age at the time of diagnosis, sex of acute diarrhea children in the ≤5 years old, all of which were then analyzed. The incidence was collected on a monthly basis. This time interval was chosen as being likely to indicate any seasonal trends or variations. 

Fecal suspensions were diluted in 0.01 M phosphate buffer saline (w/v) (pH 7.2), vortexed, and clarified through centrifugation at 10,000*g *for 20 min to a final concentration of 10% w/v. The samples were subjected to total nucleic acid extraction using a QIA Viral RNA Mini Kit (QIAGEN, Hilden, Germany) according to the manufacturer's instructions. Viral nucleic acids were eluted in 50 μl of Buffer AVE with their DNA or RNA extracted, and stored at −80°C until analyzed using molecular methods.

**Viral detection**

Reverse transcription (RT) was detected for the presence of RNA viruses. Briefly, all reverse transcription reactions were performed using the RevertAid^TM^ First Strand cDNA Synthesis kit (Fermentas GmbH, Germany). The RT reaction mix was prepared on an ice rack by combining 8 μL total RNA, 1 μL of 0.2 mg/ml random Hexamer, and 2μL nuclease-free water. The reaction mix was incubated at 70C for 3 min before the tubes were placed on ice for 2 min. Next, 4 μL of 5xRT buffer, 2 μL of 10 nM dNTP mix, 1 μL of 20 U/ml RiboLock RNase inhibitors, as well as 1 μL of 200 U/ml RevertAid M-MuLV RT, and 2μL nuclease-free were added. The reaction tubes were incubated at 42^o^C for 1 hour and then at 95^o^C for 5 min to inactivate the RTase. The final RT reaction products were stored at −20°C for further analysis. Single PCR reactions were performed from extracting DNA or CDNA (SaVs and HAstV), using primers specific to each virus previously described in Table 1 (15-17). The reaction was performed in a 25μl volume containing 10μL 2x master mix (AccuPower HF PCR PreMix, Bioneer-Korea), 0.5 μL each primer, 4μL DNA or CDNA, and 10 μL distilled water. Amplification was performed using a Veriti 96-Well Thermal Cycler (Applied Biosystems™, USA) with the Thermal cycling parameters for the PCR reactions being as follows: 94 ^°^C for 5 min followed by 40 cycles at 94 ^°^C for 45 s, 60 ^°^C for 45 s, 72 ^°^C for 45 s with a final cycle at 72 ^°^C for 5 min. The final PCR product was loaded in gel electrophoresis using 1× Tris-acetate EDTA (TAE) buffer pH 8.3 on 2% agarose gels, containing GelRed™ (Biotium, USA) and visualized under UV transilluminator apparatus (UVITEC, UK).

**Statistical methods**

All data analyses were performed using SPSS statistical software v. 19.0.1 (SPSS Inc., USA). Continuous variables were expressed as mean and standard deviation for normally distributed data, while categorical variables were expressed as frequency and percentage. A χ^2^ test was used to determine whether significant differences exist between two categorical variables. P-value less than 0.05 was considered statistically significant. 

## Results

**Figure 1:**
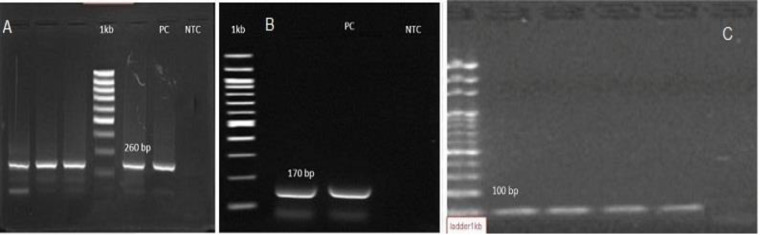
Detection of Human Astrovirus (HAstV), enteric Adenovirus (HAdV), and Sapovirus (SaV) by PCR. A) HAdV, B) HAstV, C) SaV. Ladder 1kb, NTC: Non-Template Control, PC: Positive Control

Fecal specimens from 120 patients with acute gastroenteritis were screened for detecting HAdVs, SaVs, and HAstV. The overall positive cases among the acute gastroenteritis patients were 17 out of 120 (14.1%) for at least one of the three enteric viruses tested. The mean age of patients was 36.2 ± 14.03 months (range 1–60 months). Males comprised the majority (70.6%), and most of the patients were *children* ≤*12 months* of age (52.9%). HAdV, HAstV, and SaV were detected in 5%, 6.7% and 2.5% of samples, respectively (Table 2 and Figure 1). Mixed infections were not detected in this study.

**Figure 2:**
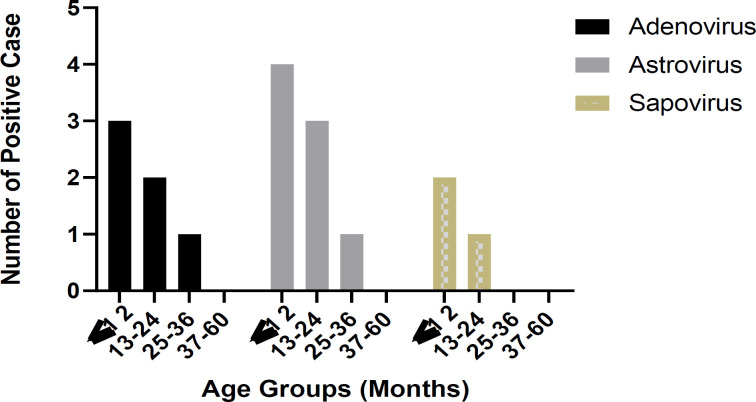
Distribution of the number of positive cases for enteric virus detection based on age groups

The age distribution of the HAdV, HAstV, and SaV positive cases is shown in Figure 2. The highest detection rate of sample positive was observed in children aged younger than 12 months compared to other old age groups (9/17, 52.9%), but there was no significant difference (P = 0.42). gNo cases were positive about HAdV, HAstV, and SaV in the > 36-month-old age group. Only two cases were positive for enteric HAdV and HAstV in the > 24-month-old age group. 

**Figure 3:**
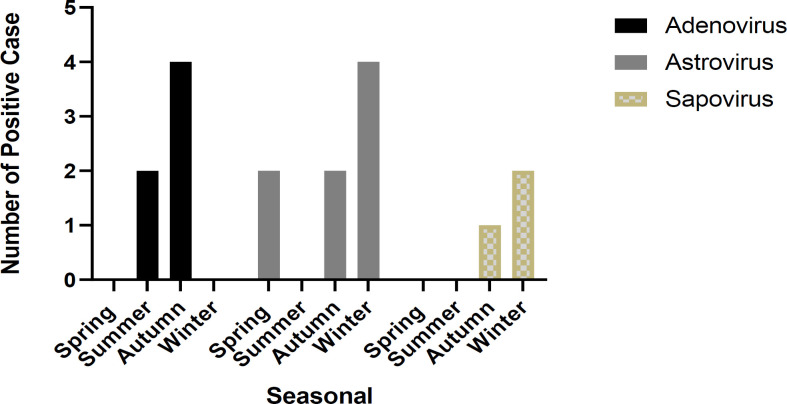
Distribution of the number of positive cases for enteric virus detection based on seasonality

The gender distribution of HAdV, HAstV, and SaV in male/female positive cases was (4/2), (6/2), and (2/1), respectively. There was no significant difference in the positive percentages of male (n = 12/17, 71%) and female children (P=0.06). Figure 3 displays the seasonal distribution of positive cases for HAdV, HAstV, and SaV observed throughout the study period. The highest rate of HAstV was identified in winter (66.7%), HAdV in fall (66.7%), and SaV in winter (33.3%), but there was no significant difference (P = 0.35). 

**Table 1:** List of specific primers for Human Astrovirus (HAstV), enteric Adenovirus (HAdV), and Sapovirus (SaV)

	Sequence	Length	Reference
HAdV	GCCACCGABACGTACTTCAGYCTG	261bp	15
	GGCRGTGCCGGAGTAGGGTTTRAA		
HAstV	TCT YAT AGA CCG YAT TAT	170bp	16
	TCA AAT TCY ACA TCR TCA CCA A		
SaV	CAVGCT CTC GCC ACC TAC	100bp	17
	CCY TCC ATY TCA AAC ACT AWT TT		

**Table 2:** Age, gender, and enteric virus agents’ data for the acute gastroenteritis patients in pediatric medical centers in Tehran, Iran

Variables	N of cases	Positive N (%)	Negative N (%)	P value
Age groups (Month) ≤ 12 13-24 25-36 37-60	24 64 16 16	9 (37.5) 6 (9.4) 2 (12.5) 0 (0.0)	15 (62.5) 58 (90.6) 14 (87.5) 16 (100)	0.42
Gender Male Female	66 54	12 (18.2) 5 (9.3)	54 (81.8) 49 (90.7)	0.06
Infections Adenovirus Astrovirus Sapovirus Overall	120 120 120 120	6 (5%) 8 (6.7%) 3 (2.5%) 17 (14.2%)	114 (95.0) 112 (93.3) 117 (97.5) 103 (85.8)	0.12

We observed that some agents, such as Adenovirus, unlike other viral agents, can also cause diarrhea in hot seasons.

## Discussion

In this study, HAstV was reported for the majority of the positive cases detected in 6.7% (8/120) of the gastrointestinal infections in children fewer than 5 years. This prevalence is higher than the prevalence of HAstV infection in most countries in the world (7, 18). In a study in Spain, a prevalence rate of 13.3% (79/330) was found for HAstV infection (8). The prevalence HAstV in males (6/8) was higher than in females. This finding is in agreement with studies conducted in Nigeria and Saudi Arabia (19, 20). In this study, the highest frequency of HAstV infection was observed among children up to 12 months and there was no positive case among >36-month-old individuals. This result is consistent with earlier studies, where the highest frequency of HAstV infection was among children before 12 months of age (19). The seasonal pattern of HAstV distribution documented in our study show a higher frequency in winter and rainy seasons, which is consistent with a majority of other studies in populations living in temperate regions (20). However, some reports exist describing higher HAstV prevalence during spring and summer months (20, 21). HAdV has been detected in previous studies in developing and developed countries as ranging from 2-35 % (22). In the present study, the percentage of positive cases was 5% which was similar to enteric HAdV prevalence (5.7%) in children with acute gastroenteritis in Iran (22, 23).

Consistent with an earlier study, the highest frequency of HAdV infection was in children ≤12 months old and >83% occurred among children ≤2 years of age (24). In some reports, there was no seasonal pattern or any peak of frequency of HAdV throughout the year (6). However, In this study, notable seasonal distribution was found, with 4 out of 6 (66.7%) HAdV cases found in fall and two cases (33.3) in summer. In this study, the rate of HAdV was twice as large in male cases compared with the females, which is in parallel with the Rezaei study report (25). Further, a previous report on Baghdad revealed that HAdV was detected 58% in males as compared to 42% in females (26). We observed all of the positive cases (2.5%) were children under 2 years of age for SaVs. Meanwhile, some studies detected a prevalence rate of 2.1% for SaVs in provinces of Iran (27). Our result was consistent with the reports showing its prevalence is usually far lower than that of HAstV and HAdV (11, 28). Also, the age distribution was similar to a previous study (11). In contrast with Varela et al. in 2019, the highest SaV prevalence was observed during the cold months (winter, fall) (29, 30). The present data showed seasonal fluctuations for prevalence of acute gastroenteritis due to Adenovirus, Astrovirus, and Sapovirus, but there was no statistical difference across various seasons for each enteric virus. 

The reason that diarrhea-associated viral infections are far more likely to occur to younger children may be the immature immune function of infants and differences in the intestinal microbiome, which changes from the first few years of life (31). To the best of our knowledge, this is the only study in Iran in which at least three enteric viruses as causative agents of diarrhea were detected simultaneously. Although the number of samples examined for this study was small, this data highlights the importance of diagnostic screening as a routine service of viral diarrhea. Also, these findings underscore the importance of monitoring the epidemiology of these viral diarrhea infections.
